# Supplementary material for: Assessing Knowledge, Beliefs, and Behaviors around Antibiotic Usage and Antibiotic Resistance among UK Veterinary Students: A Multi-Site, Cross-Sectional Survey
Source: Antibiotics (Basel). 2022 Feb 16;11(2):256. doi: 10.3390/antibiotics11020256 (PMC8868437; doi:10.3390/antibiotics11020256)
Supplement: Supplementary file 1 [file antibiotics-11-00256-s001.zip › antibiotics-1542519-supplementary.pdf]

Supplemental Materials

Table S1. Demographic variables for whole sample by study year and significance checks for group differences.

	Number of Participants per Study Year (Percentage of Sample)				Test of Difference		
**Measure**	**First (*n* = 260)**	**Second (*n* = 200)**	**Third (*n* = 60) [2]**	**Fourth (*n* = 53) [2]**	**Fisher's Exact Test**	***p***	***S***
**Age (Years; *n* = 573)**					15.14	.71	0.49
18 - 24	240 (45.6)	186 (35.4)	53 (10.1)	47 (8.9)			
25 – 34 [1]	15 (40.5)	11 (29.7)	6 (16.2)	5 (13.5)			
35 - 44	2 (50.0)	1 (25.0)	1 (25.0)	0 (0.0)			
45 – 54	1 (100.0)	0 (0.0)	0 (0.0)	0 (0.0)			
55 - 64	1 (50.0)	0 (0.0)	0 (0.0)	1 (50.0)			
65 and Over	0 (0.0)	1 (100.0)	0 (0.0)	0 (0.0)			
Prefer Not to Say	1 (50.0)	1 (50.0)	0 (0.0)	0 (0.0)			
**Gender (*n* = 573)**					8.82	.44	1.18
Female	216 (46.1)	167 (35.6)	46 (9.8)	40 (8.5)			
Male [1]	42 (42.4)	30 (30.3)	14 (14.1)	13 (13.1)			
Other	1 (100.0)	0 (0.0)	0 (0.0)	0 (0.0)			
Prefer Not to Say	1 (25.0)	3 (75.0)	0 (0.0)	0 (0.0)			
**Ethnicity (*n* = 573)**					16.53	.26	1.94
White [1]	230 (44.6)	185 (35.9)	55 (10.7)	46 (8.9)			
Black	1 (100.0)	0 (0.0)	0 (0.0)	0 (0.0)			
Asian [1]	13 (56.5)	5 (21.7)	2 (8.7)	3 (13.0)			
Mixed	15 (57.7)	7 (26.9)	3 (11.5)	1 (3.8)			
Other	0 (0.0)	1 (33.3)	0 (0.0)	2 (66.7)			
Prefer Not to Say	1 (25.00)	2 (50.0)	0 (0.0)	1 (25.0)			

Note. [1] Percentages do not exactly total 100% due to rounding. [2] Participants only recruited from University of Surrey in these cohorts

Table S2. Demographic variables for whole sample by university and significance checks for group differences

	Number of Participants per University (Percentage of Sample)			Test of Difference		
**Measure**	**Bristol (*n* = 237)**	**Liverpool (*n* = 65)**	**Surrey (*n* = 271)**	**Fisher's Exact Test**	***p***	***S***
**Age (Years; *n* = 573)**				13.44	.24	2.06
18 - 24	224 (42.6)	61 (11.6)	241 (45.8)			
25 - 34	11 (29.7)	4 (10.8)	22 (59.5)			
35 - 44	0 (0.0)	0 (0.0)	4 (100.0)			
45 - 54	1 (100.0)	0 (0.0)	0 (0.0)			
55 - 64	0 (0.0)	0 (0.0)	2 (100.0)			
65 and Over	1 (100.0)	0 (0.0)	0 (0.0)			
Prefer Not to Say	0 (0.0)	0 (0.0)	2 (100.0)			
**Gender (*n* = 573)**				3.11	.89	0.17
Female	193 (41.2)	56 (11.9)	220 (46.9)			
Male	43 (43.4)	9 (9.1)	47 (47.5)			
Other	0 (0.0)	0 (0.0)	1 (100.0)			
Prefer Not to Say	1 (25.0)	0 (0.0)	3 (75.0)			
**Ethnicity (*n* = 573)**				8.14	.60	0.74
White	214 (41.5)	59 (11.4)	243 (47.1)			
Black	0 (0.0)	0 (0.0)	1 (100.0)			
Asian	13 (56.5)	2 (8.7)	8 (34.8)			
Mixed [1]	9 (34.6)	3 (11.5)	14 (53.8)			
Other	0 (0.0)	0 (0.0)	3 (100.0)			
Prefer Not to Say	1 (25.0)	1 (25.0)	2 (50.0)			
**Year of Study (*n* = 460)**				2.31 [2]	.31	1.69
First	126 (48.5)	38 (14.6)	96 (36.9)			
Second	111 (55.5)	27 (13.5)	62 (31.0)			

Note. [1] Percentages do not exactly total 100% due to rounding. [2] Value for Pearson's chi-square test reported

Tests of Difference for Beliefs

Beliefs about responsibility for causing ABR – early years.

There were no statistically significant differences in beliefs between universities on three subscales, but there were statistically significant differences in beliefs for the Public/Patient subscale (see Table S3). These were explored using pairwise comparisons with adjusted p values. There were statistically significant differences between Surrey and Bristol students, but not between Surrey and Liverpool students, or between Liverpool and Bristol students. Effect sizes were small, but compared to Bristol early years students, Surrey early years students had higher mean beliefs that 1) public/patients have responsibility for causing ABR, $p = .003$, $S = 8.38$, $r = .17$ (Surrey versus Liverpool, $p = .067$, $S = 3.90$, $r = .16$; Bristol versus Liverpool, $p = 1.00$, $S = 0.00$, $r = .002$)

Table S3. Kruskal-Wallis tests for differences in mean beliefs (responsibility for causing ABR) for whole early years sample between universities ($n = 436$)

Measure	H (df)	p	S
Human Medics	1.22 (2)	.54	0.89
Public/Patients	11.71 (2)	.003	8.38
Vets	2.01 (2)	.37	1.43
Animal Owners	5.55 (2)	.062	4.01

Note. df = Degrees of freedom.

Beliefs about responsibility for preventing ABR – early years.

There were no statistically significant differences in beliefs between universities on two subscales, but there were statistically significant differences in beliefs for the Public/Patients subscale and the Animal Owners subscale (see Table S4). These were explored using pairwise comparisons with adjusted p values. For both subscales, there were statistically significant differences between Surrey and Bristol students, but not between Surrey and Liverpool students, or between Liverpool and Bristol students. Effect sizes were small, but compared to Bristol early years students, Surrey early years students had higher mean beliefs that 1) public/patients have responsibility for preventing ABR, $p = .044$, $S = 4.51$, $r = .13$ (Surrey versus Liverpool, $p = 1.00$, $S = 0.00$, $r = .04$; Bristol versus Liverpool, $p = .83$, $S = 0.27$, $r = .06$), and 2) animal owners have responsibility for preventing ABR, $p = .012$, $S = 6.38$, $r = .15$ (Surrey versus Liverpool, $p = .22$, $S = 2.18$, $r = .13$; Bristol versus Liverpool, $p = 1.00$, $S = 0.00$, $r = .01$).

Table S4. Kruskal-Wallis tests for differences in mean beliefs (responsibility for preventing ABR) for whole early years sample between universities ($n = 435$)

Measure	H (df)	p	S
Human Medics	4.43 (2)	.11	3.18
Public/Patients	6.13 (2)	.047	4.41
Vets	1.30 (2)	.52	0.94
Animal Owners	8.64 (2)	.013	6.27

Note. df = Degrees of freedom.

Beliefs among early years and later years.

Beliefs about the amount of responsibility the four groups have for both causing and preventing ABR were not observed to statistically significantly differ between early and later years vet students at Surrey, with the exception of the Animal Owners subscale on the preventing ABR scale (see Table S5).

Table S5. Mann-Whitney tests for differences in beliefs between early and later years University of Surrey students

Measure	*U*	*z*	*p*	*S*	*r*
**Responsibility for Causing ABR (n = 253)**					
Human Medics	6,931.50	1.61	.11	3.18	.10
Public/Patients	7,215.50	1.12	.27	1.89	.07
Vets	7,241.50	1.07	.29	1.79	.07
Animal Owners	7,307.50	0.95	.34	1.56	.06
**Responsibility for Preventing ABR (n = 251)**					
Human Medics	7,796.00	0.13	.90	0.15	.008
Public/Patients	7,165.50	1.00	.32	0.00	.06
Vets	8,389.50	1.19	.24	2.06	.08
Animal Owners	6,283.50	2.67	.008	6.97	.17
